# Does the internal jugular vein affect the elasticity of the common carotid artery?

**DOI:** 10.1186/s12947-016-0084-1

**Published:** 2016-09-17

**Authors:** Michał Podgórski, Monika Winnicka, Michał Polguj, Piotr Grzelak, Maciej Łukaszewski, Ludomir Stefańczyk

**Affiliations:** 1 Department of Radiology and Diagnostic Imaging, Medical University of Lodz, 22, Kopcińskiego St., Barlicki Hospital, Lodz, Poland; 2 Department of Angiology, Chair of Anatomy, Medical University of Lodz, 60, Narutowicza St, Lodz, Poland; 3 Department of Diagnostic Imaging, Polish Mother’s Memorial Hospital Research Institute, 281/289, Rzgowska St, Lodz, Poland

**Keywords:** Atherosclerosis, Arterial stiffness, 2D-Speckle tracking, β-Stiffness index, Carotid artery

## Abstract

**Background:**

Arterial stiffness is an early marker of atherosclerosis. The carotid arteries are easily accessible by ultrasound and are commonly used for the evaluation of atherosclerosis development. However, this stiffness assessment is based on the elastic properties of the artery, which may be influenced by the adjacent internal jugular vein (IJV).

The aim of the present study is to evaluate the influence of internal jugular vein morphology on the stiffness of the common carotid artery.

**Methods:**

Bilateral carotid ultrasound was performed in 248 individuals. When no carotid plaque was detected (90.9 % cases), the distensibility coefficient and β - stiffness index were calculated. The global and segmental circumferential strain parameters of the carotid wall were evaluated with 2D-Speckle Tracking. The cross-sectional area of the IJV and degree of its adherence to the carotid wall (angle of adherence) were measured.

**Results:**

The morphology of the IJV did not influence the standard stiffness parameters nor the global circumferential strain. However, segmental analysis found the sector adjacent to the IJV to have significantly higher strain parameters than its opposite counterpart. In addition, the strain correlated significantly and positively with IJV cross-sectional area and angle of adherence.

**Conclusions:**

The movement of the carotid artery wall caused by the passage of the pulse wave is not homogeneous. The greatest strain is observed in a segment adjacent to the IJV, and the degree of wall deformation is associated with the size of the vein and the degree of its adherence.

## Background

Although loss of arterial elasticity naturally occurs with ageing, it is also an early marker of atherosclerosis. The pathomechanism of arterial stiffening is associated mostly with the exchange of elastin for collagen in the extracellular matrix of the arterial wall [1, 2]. Such structural changes have a strong impact on the generation, propagation and reflection of pressure waves in the arterial tree, resulting in an increased aortic systolic pressure, a greater burden on the left ventricle and increased risk of cardiovascular mortality [2, 3].

Arterial stiffness may be assessed through such systemic markers as pulse wave velocity or augmentation index, or locally in parts of the cardiovascular system most prone to development of atherosclerosis [4, 5]. Commonly-used markers of local arterial stiffness are distensibility coefficient, elastic modulus and β-stiffness index [2, 4–6]. A newly-developed method derived from echocardiography which can be employed in the evaluation of local arterial stiffness is 2D-Speckle Tracking [3]. This tool offers the advantage of a more detailed, segment-base analysis of arterial wall mechanics than standard stiffness parameters [3, 7]. Moreover, unlike tissue Doppler imaging and IMT measurements, it is angle independent [8]. It is also more sensitive than β-stiffness index in the detection of age-related changes in the arterial wall elasticity [5]. 2D-Speckle Tracking might therefore be useful in determining whether local conditions may influence the elastic properties of the arterial wall and bias any stiffness assessment.

The internal jugular vein (IJV) travels adjacent to the internal carotid and common carotid arteries (CCA) within the carotid sheath. Its size and course are highly variable, nevertheless it is easily compressible due to its thin wall and low blood pressure [9, 10]. Hence, it is reasonable to assume that the elasticity of the common carotid artery may be affected by the size and alignment of the IJV.

Hence, the aim of the study was to evaluate the influence of IJV morphology on stiffness markers evaluated in the CCA.

## Methods

Carotid ultrasound was performed in 248 participants of the “Diamentowy Grant” study (No DI2012 007742), the aim of which was to assess the relationship between asthma and risk of atherosclerosis. All participants gave their informed consent to take part in the study, and the study protocol was approved by the Local Bioethics Committee (RNN/41/13/KB).

Patients were recruited from the Pulmonology and Allergology Outpatient Clinics and through an internet advertisement. The only inclusion criterion was that the participant must be aged over 30 years old. The exclusion criteria were as follows: the presence of atrial fibrillation, which impair the evaluation of strain parameters, the presence of goitre or lymphadenopathy adjacent to CCA or IJV, or previous surgeries in the neck region. When the atherosclerotic plaque was present in the CCA or its bifurcation, this side was excused from the analysis.

### Examination

Carotid ultrasound was performed with a GE Vivid 7 ultrasound apparatus (GE Medical System, Milwaukee, WI, USA) with a high-resolution linear transducer (14 MHz).

The patient lay in the supine position. After 5 min rest under semi-dark, quiet conditions, brachial blood pressure was measured, an ECG trace was obtained and carotid ultrasound was performed. The patient’s head was turned 45° opposite to the side of examination. The carotid arteries were evaluated for the presence of atherosclerotic changes. If no changes were noted, the short axis of the CCA was obtained one centimetre below the carotid bulb. Any movement between the two most distant points on the near and far walls of CCA was assessed using M-mode during three consecutive heart cycles. Afterwards, the short axis of the CCA was visualised in standard B-mode and a cine loop taken during another three consecutive heart cycles was saved. If the entire IJV did not fit within the field of view, another three consecutive heart cycle cine loop was recorded to see a complete cross-section of the IJV.

To minimize respiration-related motion artefacts, all acquisitions were performed during a short breath-hold at the end of expiration. The probe was placed with the least possible pressure to avoid compressing the IJV and to allow expansion of the CCA. All images were recorded with a high frame rate (>90 frames s^−1^; mean frame rate: 112 ± 20 frames s^−1^).

Further analysis was performed offline on a workstation equipped with EchoPac software (EchoPac PC, GE Medical System). The measurements from three cardiac cycles were averaged and used for further analysis. Based on the M-mode presentation, the classical arterial stiffness parameters were calculated according to the following formulas:

1. Distensibility coefficient (DC)
  $$ DC\left[P{a}^{-1}\right]=\frac{\left({D}_{\max}^2-{D}_{\min}^2\right)}{{D_{\min}}^2}\times \varDelta P $$
  ∆P difference between systolic and diastolic blood pressure value; D_max_ and D_min_ are respectively the largest and the smallest distances between the intima media thickness on the near and far wall of the CCA.
2. β-stiffness index (β)
  $$ \beta =\frac{ \ln \left(\frac{SBP}{DBP}\right)\times D}{\varDelta D} $$
  SBP - systolic blood pressure, DBP - diastolic blood pressure, D - mean value of D_max_ and D_min_, ΔD - difference between D_max_ and D_min_

Circumferential strain (CS) and strain rate (CSR) were evaluated using 2D-Speckle Tracking. The region of interest (ROI) was placed over the arterial wall along the border between the intima-media and the vessel lumen. The width of the ROI was narrower to cover the smallest possible portion of tissues adjacent to the arterial wall. The ROI segments were manually adjusted so that one of them covered the whole part of the CCA wall adherent to the IJV wall (venous segment). A mirror segment of the same length was placed against the opposite site (opposite segment). The parts of the wall between these segments were covered with remaining two parts of the ROI – the posterior and anterior segments. “Global” and “segmental” values of CS and CSR were calculated as the mean amplitudes between minimal and maximal measurements during the three heart beats of the cine loop.

**Fig. 1:**
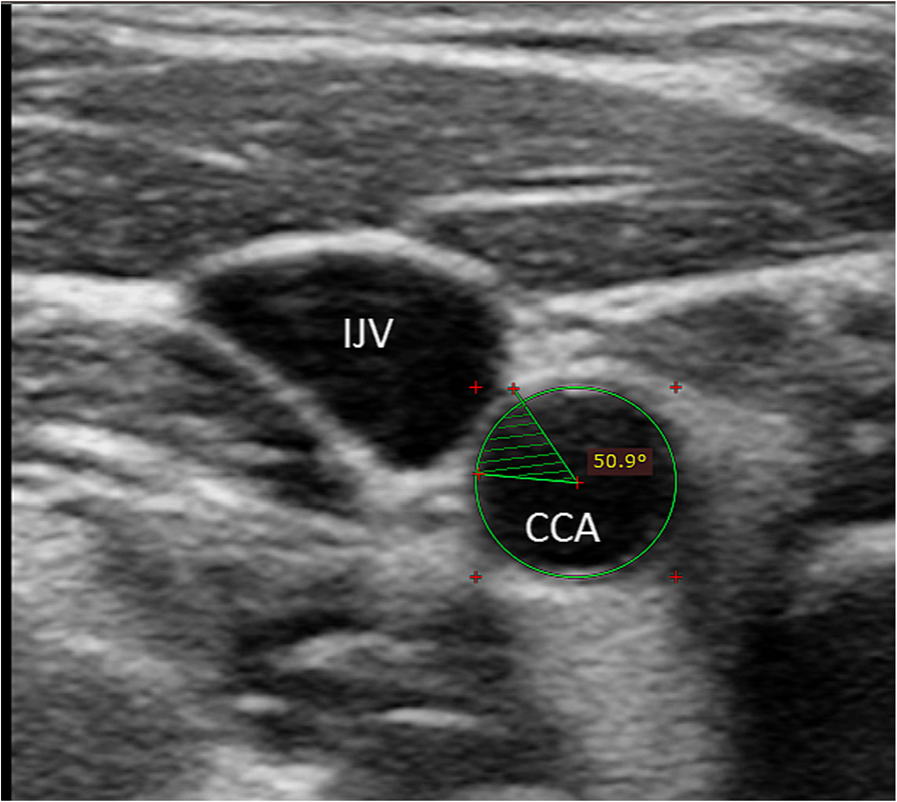
Example of the “angle of adherence” measurement. CCA - common carotid artery; IJV – internal jugular vein

The cross-sectional area of the CCA and the IJV were measured with a tracking-measuring tool. If the CCA and IJV were not in contact, the distance between them was measured. If they were in contact, the segment including CCA and IJV adjacent to each other was measured as a portion of the complete circumference of the CCA in degrees and this was determined the “angle of adherence” (Fig. 1).

### Statistical analysis

The statistical analysis was performed using Statistica 12 software (StatSoft Polska, Cracow, Poland). A *p*-value lower than 0.05 was considered significant. The results are presented as mean and standard deviation unless otherwise stated.

The normality of the continuous data distribution was checked with the Shapiro-Wilk test. The χ2 test was used for comparisons of nominal variables. Comparisons of continuous variables between different groups (e.g. men vs women) were performed with the Student *t*-test for independent variables. Differences in continuous variables between body sides was performed with the paired Student *t*-test. To evaluate the determinants of stiffness, multiple regression analysis was performed with age, BMI, systolic blood pressure, angle of adherence and cross-sectional area of the IJV, as potential explanatory variables. The correlation of continuous variables was assessed with the Persons correlation coefficient. Our previous studies have confirmed the reliability of strain measurements to be 84.83 % for interclass and 94.42 % for intraclass agreement [11].

## Results

A carotid plaque was found on 25 left CCA and 20 right CCA. In five individuals, it was present bilaterally. Hence, 223 left CCA and 228 right CCA were included into the analysis: 90.9 % from all arteries. In the study group there were 66 (27 %) men and 177 (73 %) women, in the mean age of 57.2 (SD = 9.3) and 56.4 (SD = 9.4), respectively. The age difference was not significant (*p* = 0.5562).

The wall of the IJV was in contact with the wall of the CCA in 212 cases on the right side (93 %) and in 213 on the left side (96 %). In remaining cases, the vessels were separated by a mean distance of 6.2 mm (SD = 5.4) on the right side and 5.9 (SD = 4.8) on the left side. The difference was not significant (*p* = 0.81).

The diameters of the vessels are presented in Table 1. Both CCA and IJV were significantly larger on the right side. The mean angle of adherence was 69.9° (SD = 36.8°) on the right side and 74.8° (SD = 34.7°) on the left side. The difference was not significant (*p* = 0.5821).

**Table 1:** Measurements of common carotid arteries and internal jugular veins. Data presented as mean and (SD)

	RCCA	LCCA	*p*	RIJV	LIJV	*p*
AP diameter [mm]	7.6 (1.0)	7.4 (0.8)	**0.0001**	5.2 (3.1)	4.6 (2.6)	**0.0036**
ML diameter [mm]	7.7 (1.0)	7.5 (0.9)	**0.0001**	11.0 (3.9)	9.8 (3.9)	**0.0001**
Area [mm^2^]	47.0 (1.0)	44.1 (1.1)	**0.0001**	55.9 (5.8)	41.2 (3.8)	**0.0001**

*RCCA* right common carotid artery, *LCCA* left common carotid artery, *RIJV* right internal jugular vein, *LIJV* left internal jugular vein, *AP* antero-posterior diameter, *ML* medio-lateral diameter

*p*-value presented in bold style is significant (<0.05)

### Global stiffness analysis

Arterial stiffness parameters are presented in Table 2. The global CSR of the right CCA correlated significantly with the area of the right IJV. The remaining parameters did not significantly correlate with the angle of adherence nor with the area of the vein (Table 3). Furthermore, multiple regression analysis found all of the arterial stiffness parameters to be independent of vein area and adherence angle (Table 4).

**Table 2:** Strain parameters for carotid arteries

	RCCA	LCCA	*p*
β - stiffness index	9.7 (4.6)	9.9 (5.1)	0.6028
Distensibility coefficient	0.06 (0.08)	0.07 (0.03)	0.2099
CS [%]	3.32 (1.34)	3.21 (1.21)	0.8319
CSR [1/s]	0.63 (0.22)	0.61 (0.21)	0.3952

*RCCA* right common carotid artery, *LCCA* left common carotid artery, β β-stiffness index, *DC* distensibility coefficient, *CS* circumferential strain, *CSr* circumferential strain rate

**Table 3:** Correlation between arterial stiffness parameters and vein related variables

		RIJV						LIJV			
		Angle		Area				Angle		Area	
		*R* ^2^	*p*	*R* ^2^	*p*			*R* ^2^	*p*	*R* ^2^	*p*
RCCA	β	0.04	0.6760	0.14	0.0770	LCCA	β	−0.10	0.2824	−0.01	0.8750
	DC	−0.02	0.8593	−0.15	0.0686		DC	0.07	0.4096	−0.03	0.6854
	CS [%]	0.01	0.9482	−0.10	0.1831		CS [%]	0.10	0.2180	−0.06	0.4054
	CSR [1/s]	−0.13	0.1005	**−0.21**	**0.0050**		CSR [1/s]	0.05	0.5061	−0.14	0.0634

*RCCA* right common carotid artery, *LCCA* left common carotid artery, *RIJV* right internal jugular vein, *LIJV* left internal jugular vein, β β-stiffness index, *DC* distensibility coefficient, *CS* circumferential strain, *CSr* circumferential strain rate, *R* ^*2*^ correlation coefficient, *p* value

*p*-value presented in bold style is significant (<0.05)

**Table 4:** Multiple regression analysis for arterial stiffness parameters (pooled data for right and left CCA)

	β *R* ^*2*^ = 0.1054 *p* = 0.005		DC *R* ^2^ = 0.0855 *p* = 0.005		CS [%] *R* ^2^ = 0.1196 *p* = 0.002		CSR [1/s] *R* ^2^ = 0.2445 *p* < 0.001	
	Beta	*p*	Beta	*p*	Beta	*p*	Beta	*p*
Age [years]	**0.154**	**0.0006**	**−0.001**	**0.0008**	**−0.053**	**0.0000**	**−0.011**	**0.0000**
BMI [kg/m^2^]	0.137	0.0903	−0.001	0.1793	−0.036	0.0934	**−0.007**	**0.0487**
SBP	−0.002	0.9170	0.000	0.0897	**0.009**	**0.0455**	**0.002**	**0.0213**
Angle of adherence	−0.012	0.2695	0.000	0.1775	0.004	0.2177	0.000	0.7683
Area of the RIJV	0.004	0.6777	0.000	0.2857	0.001	0.6520	0.000	0.7618

*RCCA* right common carotid artery, *LCCA* left common carotid artery, *RIJV* right internal jugular vein, *LIJV* left internal jugular vein, β β-stiffness index, *DC* distensibility coefficient, *CS* circumferential strain, *CSr* circumferential strain rate, *BMI* body mass index, *SBP* systolic blood pressure, *Beta* regression coefficient, *p* – value

*p*-value presented in bold style is significant (<0.05)

### Segment specific analysis

**Fig. 2:**
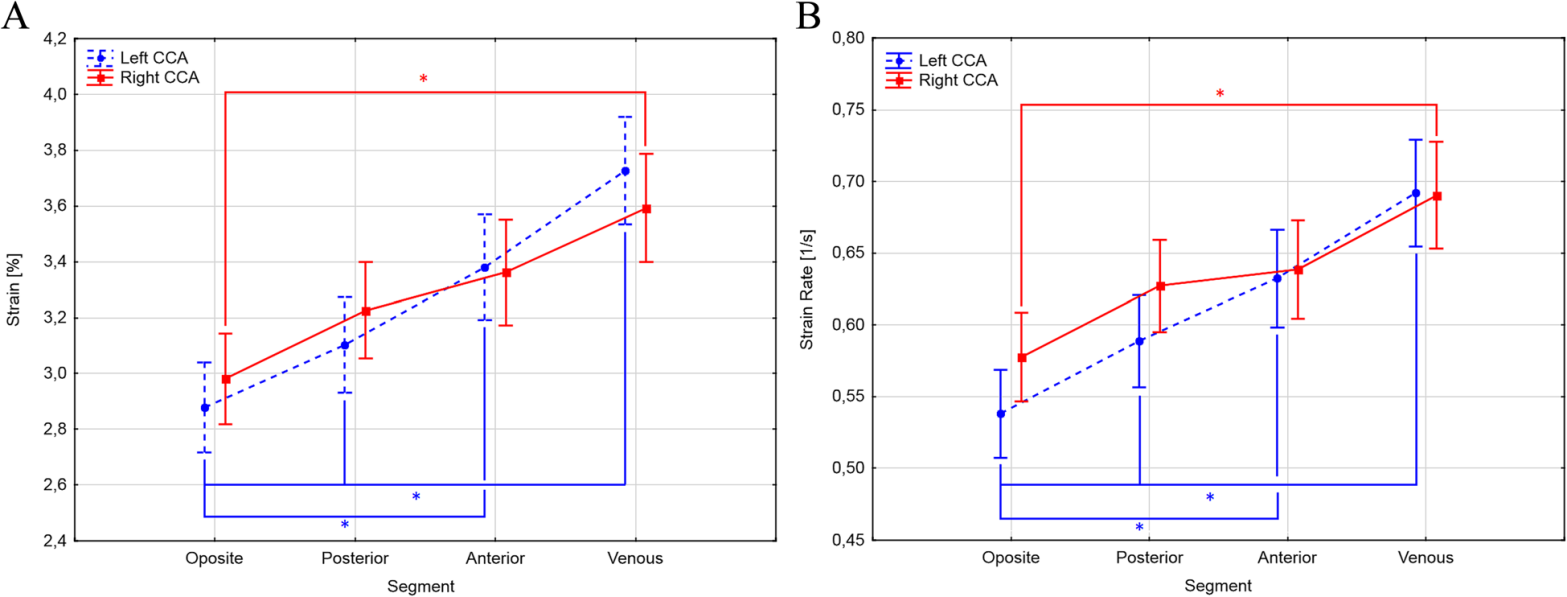
Plot depicting post-hoc comparison of circumferential strain (**a**) and circumferential strain rate (**b**) between segments of the right and left common carotid artery. Central point represents mean and whiskers a standard deviation. * - significant difference; CCA - common carotid artery

Both CS and CSR differed significantly between the analysed segments (Table 5). For the LCCA, the CS and CSR of the venous segment were significantly higher than of the opposite and posterior segments. These parameters were also significantly higher in the anterior segment than in the opposite segment (Fig 2.).

**Table 5:** Differences in circumferential strain and circumferential strain rate according to the analysed segment of the common carotid artery

Segment	LCCA		RCCA	
	CS [%] mean (SD)	CSr [1/s] mean (SD)	CS [%] mean (SD)	CSr [1/s] mean (SD)
Opposite	2.88 (1.25)	0.54 (0.24)	2.98 (1.32)	0.64 (0.26)
Posterior	3.10 (1.26)	0.59 (0.24)	3.23 (1.39)	0.58 (0.23)
Anterior	3.38 (1.43)	0.63 (0.27)	3.36 (1.48)	0.63 (0.25)
Venous	3.73 (1.57)	0.69 (0.29)	3.59 (1.56)	0.69 (0.28)
*p*	**<0.0001**	**<0.0001**	**0.0012**	**0.0017**

*RCCA* right common carotid artery, *LCCA* left common carotid artery, *CS* circumferential strain, *CSr* circumferential strain rate, *p* – value

*p*-value presented in bold style is significant (<0.05)

The CS and CSR of the RCCA were significantly higher only when the venous segment was compared with the opposite one (Fig 2.).

The CS and CSR of the venous segment correlated significantly with the area of the IJV cross-section (LCCA: strain - *R* ^2^ = 0.51, *p* = 0.0031; strain rate - *R* ^2^ = 0.48, *p* = 0.0075. RCCA: strain - *R* ^2^ = 0.53, *p* = 0.0021; strain rate - *R* ^2^ = 0.50, *p* = 0.0125) and angle of its adherence (LCCA: strain - *R* ^2^ = 0.43, *p* = 0.0078; strain rate - *R* ^2^ = 0.40, *p* = 0.0097. RCCA: strain - *R* ^2^ = 0.49, *p* = 0.0010; strain rate - *R* ^2^ = 0.47, *p* = 0.0202). The later correlation was not as tight.

## Discussion

Our findings indicate that the local elasticity of the CCA is affected by the adherence of the IJV. Although it does not seem to affect the global elastic properties of the artery, it may bias one-dimensional measurement of elastic properties of the CCA.

It has been known for 50 years that the rigidity of the capillary vessel is largely affected by surrounding tissue according to the “tunnel-in-gel” concept [12]. This theory has also been confirmed in a pig animal model for carotid and femoral arteries. Liu [13] notes that the CSR was found to be 15 to 25 % less when subjected to radial constraint at physiological pressure. In addition, mean circumferential wall stress only constituted a maximum of 30 % of the untethered stress. Asymmetrical expansion of the carotid artery has also been reported in a rat model based on longitudinal sections of the CCA and external carotid artery [14]. However, no detailed analysis of neighbouring structures was included in any of the aforementioned experiments.

The local measurement of arterial stiffness reveals the relationship between changes in the arterial volume and distending pressure [2]. The calculation assumes that the luminal cross-sectional area changes linearly with pressure and that the length of the artery remains constant during contraction [2]. However, as luminal distensibility of the arterial wall is not representative of whole arterial wall stress, calculation of the β-stiffness index and distensibility coefficient produces inaccurate results [15], as demonstrated by the significant differences found in segmental strain parameters in the present study. The non-homogeneous pattern of arterial stiffening is reflected in the local formation of atherosclerotic plaques which favours the posterior wall of the internal carotid artery [14]. This is in line with our results, because the opposite segment, usually comprising the posterior wall, was characterised by the least local elasticity, and so would be the most prone to plaque formation due to greater shearing stress.

2D-Speckle Tracking has been reported to offer excellent reproducibility when evaluating patients with subclinical atherosclerosis [3, 5, 15]. It gives better reproducibility when assessing arterial stiffness based on classical parameters [3, 7, 8]. This technique enables angle-independent calculations to be performed, which is especially important for operator-dependant ultrasound examination [4, 5, 7]. In addition, it has been found to be more sensitive than elastic modulus and β-stiffness index in detecting age-related differences in the elastic properties of CCA [5, 7]. In majority of studies, the global or far wall segment CS parameters were calculated because they offer better reproducibility than an analysis of each particular segment separately [2, 7]. Although one previous study, including 51 healthy subjects [8], has used bilateral segment-based analysis, it only reported significant variation in the anterior and inferior segments of the left CCA, the segments were determined automatically and their alignment was not adjusted for the neighbouring IJV.

Our findings indicate that movement of the carotid artery wall due to the passage of the pulse wave is non-homogeneous. Therefore, the evaluation of CCA diameter in a fixed manner, as the distance between a near and far wall, may not be as accurate as using 2D-Speckle Tracking to evaluate changes in diameter. Hence, standard stiffness parameters calculated based on routine measurements might be biased by the cross-sectional area of the IJV and the angle of its adherence to the CCA.

The potential limitation of this study is that it focuses only on the immediate neighbourhood of the IJV and not all surrounding tissues: It is possible that changes in the composition of loose connective tissue within the carotid sheath might also affect strain parameters. However, the aim of this research was to evaluate local (segmental) differences, and not the influence of homogeneous surroundings. The IJV was the most significant “soft point” adjacent to the CCA wall, which may increase its local elasticity. Secondly, assessment of brachial pressure instead of carotid pressure might have biased evaluation of stiffness parameters [6]. However, this effect is particularly pronounced in young subjects, when in our study the mean age of participants was 56 years. Furthermore, application of brachial pressure may lead to overestimation of stiffness parameters. Nevertheless, it might have increased the chance of significant relations between these parameters and internal jugular vein morphology but the results were not significant. Finally,, the study does not evaluate the actual error of standard measurements due to variations in IJV morphology. However, as it is now known that the IJV does play a role, this factor should be taken into account when planning further studies incorporating more advanced techniques, such as MRI.

## Conclusion

This is the first report to indicate that IJV morphology has a direct influence on strain parameters of the CCA. Due to increasing role of stiffness parameters as markers of atherosclerosis and surrogates of cardiovascular events, their evaluation should be accurate. 2D-Speckle Tracking is a sensitive and reliable method that allows for evaluation of the complete circumference of the CCA including the influence of IJV position. Hence, it offers the potential to become a superior tool for conventional stiffness measurements.

## Abbreviations

2D: Two-dimensional
CCA: Common carotid artery
CS: Circumferential strain
CSr: Circumferential strain rate
D: Mean value of D_max_ and D_min_
DBP: Diastolic blood pressure
DC: Distensibility coefficient
D_max_: The largest distances between the intima media thickness on the near and far wall of the CCA
D_min_: The smallest distances between the intima media thickness on the near and far wall of the CCA
IJV: Internal jugular vein
MRI: Magnetic resonance imaging
ROI: Region of interest
SBP: Systolic blood pressure
SD: Standard deviation
ΔD: Difference between D_max_ and D_min_
